# Diagnostic accuracy of radiography techniques for musculoskeletal disorders

**DOI:** 10.6026/973206300221444

**Published:** 2026-03-31

**Authors:** Rishabh Yadav, Rinku Choudhury, Abhijeet Alok, Mukesh Kumar, Amrita Pandita Bhatia, Tanvi Hirani, Rahul Tiwari

**Affiliations:** 1 Department of Radiology, Prasad Medical College, Lucknow, Uttar Pradesh, India; 2 Department of General Medicine, PDU Medical College and Hospital, Churu, Rajasthan, India; 3 Department of Oral Medicine and Radiology, Sarjug Dental College and Hospital, Darbhanga, Bihar, India; 4 Department of Oral & Maxillofacial Surgery, Shree Bankey Bihari Dental College, Masuri, Ghaziabad, Uttar Pradesh, India; 5 Department of Prosthodontics (Crown and Bridges and Implantology), YCCM and RDF's Dental College and Hospital, Ahilyanagar, Maharashtra, India; 6 Department of Periodontology and Implantology, Karnavati School of Dentistry, Karnavati University, Gandhinagar, Gujarat, India; 7 Department of Oral and Maxillofacial Surgery, RKDF Dental College and Research Centre, Sarvepalli Radhakrishnan University, Bhopal, Madhya Pradesh, India

**Keywords:** Musculoskeletal disorders (MSK), radiography, tomosynthesis, computed tomography (CT), magnetic resonance imaging (MRI)

## Abstract

Conventional radiography often fails to detect early or subtle musculoskeletal pathology because of limited sensitivity and anatomical overlap. Therefore, it is of interest to study prospectively evaluated CR, DT, LDCT and MRI against a composite reference standard in a tertiary MSK unit. Hence, a total of 120 patients were included in the study, of which 54 had positive findings regarding clinically significant musculoskeletal pathology. MRI showed the highest sensitivities (98.1%) and overall accuracy (95.8%), followed by low-dose CT with sensitivity(s) of 81.5% and an accuracy of 90.0%. Tomosynthesis was superior to radiography for detection and offered a clinically useful intermediate alternative for indeterminate radiographs.

## Background:

Conventional radiography is the primary imaging modality for musculoskeletal (MSK) dysfunction, as it is readily available, fast to perform and relatively cheap; however it lacks sensitivity in the diagnosis of structural change due to anatomical overlap and poor visualisation of early cortical disruption, marrow oedema or subtle structural change. Recent improvements in MSK CT systems have made possible the use of low-dose protocols with clinically relevant spatial resolution for both fracture and osseous lesion detection [1]. Concurrently, contemporary musculoskeletal (MSK) MRI sequences offer excellent soft-tissue differentiation and have a high sensitivity in detecting early bone marrow changes, so that MRI has become an important escalation method in case of negative radiographs despite continued strong clinical suspicion [2, 3]. Imaging choice is more particularly challenging in suspected infection and chronic inflammatory diseases, where early changes might be cryptic at plain films [4]. Functional and hybrid imaging techniques play also a role in particular cases where standard imaging is equivocal [5]. Therefore, it is of interest to compare the diagnostic performance of conventional radiography, digital tomosynthesis, low-dose computed tomography and magnetic resonance imaging in detecting clinically relevant musculoskeletal pathology in routine clinical practice.

## Materials and Methods:

This study aimed to determine diagnostic accuracy in a single center, prospectively, in a tertiary care radiology department from March 2024 to January 2025. Participants over 18 years old with suspected musculoskeletal disorders (MSK) fulfilling study requirements after a first clinical evaluation were included. All patients received standard radiographs and, in accordance with clinical guidelines, were assessed with digital tomosynthesis and low-dose CT. MRI of the affected region was performed as a problem-solving tool to alleviate symptoms or to resolve any clinical suspicion that persisted. MRI findings plus clinical follow-up of at least 6 weeks, Orthopedic/ Rheumatology consensus and, when applicable, surgical findings comprised the composite reference standard. The primary outcome of the study was the evaluation of the accuracy in diagnosis of clinically relevant MSK pathology (occult fracture, early inflammatory change, clinically significant osseous lesion and infection related abnormality). Sensitivity, specificity, positive predictive value (PPV), negative predictive value (NPV) and overall accuracy were calculated for each modality. Two radiologists (≥5 years of experience) interpreted the images and were blinded to the results of the other modalities to resolve discrepancies by consensus. Statistical calculations were performed using standard formulas for diagnostic tests and the results are presented as proportions.

## Results:

A total of 120 patients were assed, who had mean age of 41.6 years with slight male predominance. Most people with the clinical red flags had no clinically relevant MSK pathology, as confirmed by reference standards (54 patients [45.0%] had clinically relevant MSK pathology and 66 [55.0%] were negative after follow-up and multidisciplinary consensus). The most common triggers for accelerated imaging were continued pain in association with negative or equivocal radiographs and a presumptive radiographic diagnosis of occult fracture or early inflammatory arthritis. This pattern of distribution corresponds to normal tertiary MSK practice where radiography is inadequate for exclusion Table 1 (see PDF). MRI had the best sensitivity (98.1%) and NPV (98.4%), thus confirming its status as the most reliable rule-out imaging modality for clinically relevant MSK pathology. LDCT reached a high specificity (97.0%) and PPV (95.7%) suggesting that while radiation is reduced, performance for detection of osseous pathology was excellent. The sensitivity was superior for digital tomosynthesis versus radiography (77.8% vs 62.9%), indicating a potential role as an intermediate escalation strategy Table 2 (see PDF).

## Discussion:

This present research found out that the traditional radiography is very specific but not sensitive to the practice of identifying clinically significant musculoskeletal pathology especially in the early stages of the disease and subtle lesions. This trend in the finding is consistent with the current evidence suggesting that radiography does not always detect occult fractures and early inflammatory or infection-related alterations; more sophisticated imaging will be required. Digital tomosynthesis has been shown to be sensitive compared to conventional radiography and with equal negative and positive predictive values, so tomosynthesis could be used as an intermediate pragmatic method in circumstances when CT or MRI are not available. Past clinical trials of suspected scaphoid injury have reported good diagnostic capability of tomosynthesis coupled with less unwarranted immobilization and diagnosis time. As far as sacroiliac joints are concerned, it has been established that tomosynthesis is superior to radiography in the detection of structural lesions without subjecting the patients to the entire dose and cost of CT via some pathways [6, 7, 8-9]. In current series, low-dose CT was well specific and positive predicting value with an overall accuracy of 90%. This is in line with the present tendency of carrying out dose optimization CT with regard to MSK care. Future projections suggest that ultra-low-dose CT can maintain up to relatively high diagnostic quality in non-displaced cases with relatively low sensitivity degradation relative to conventional CT; and this observation is similar to relative performances between modalities as reported in our studies [10, 11]. In this research, MRI still does better than any of the other imaging modalities and has close to ceiling sensitivity and negative predictive value. This confirms recent diagnostic literature which has revealed that MRI is best to be used over plain film radiographs to detect occult injury since it indicates marrow edema and evaluates of soft tissues. The meta-analysis on occult hip fractures proves that MRI is slightly superior to CT in pooled sensitivity and specificity in terms of providing support to it as a final test of escalation [12]. Although this literature review was restricted to radiography and cross-sectional imaging, out-of-hospital MSK diagnostic pathways could be wider and cover ultrasound and nuclear medicine. Ultrasound imaging may be helpful in detection of fractures or soft tissues, it depends on the operator and its diagnostic accuracy varies with the anatomical area and protocol [13]. On complex indications of infection and /or implantation, Nuclear Medicine techniques particularly play an important role (PET/CT, WBC SPECT/CT). This is similar to other recent discoveries [9] which suggest that the capacity of diagnosis of chronic periprosthetic joint infection (PJI) can be impaired in case of low grade disease when it is based on visual analysis alone, demonstrating the complementary qualities of multimodality imaging. Similarly, it is proposed that suspected low-grade infection in non-unions of the long bones through FDG PET-CT results in moderate precision and low NPV, which indicates that the absence of findings should not prevail over high clinical suspicion [14, 15]. The main limitations of the present study are a single center design and composite reference standard. However, real-world MSK workflow is pervaded by permutation of tests to enable clinical interpretation and offers performance comparison in a form open to clinical interpretation in scenario-based escalation algorithms.

## Conclusion:

The specificity of conventional radiography has been high however the most clinically relevant components of musculoskeletal pathology have been missed. Although MRI has the greatest overall diagnostic reliability, low-dose CT and digital tomosynthesis have demonstrated superior sensitivity. Clinical suspicion, access and definitive need of exclusion are the factors that predetermine the need of imaging escalation.

## Advancement to knowledge:

This study demonstrates the comparative diagnostic performance of conventional radiography, digital tomosynthesis, low-dose CT and MRI in detecting clinically significant musculoskeletal pathology in routine clinical practice. The findings suggest that digital tomosynthesis and low-dose CT provide meaningful diagnostic improvements over conventional radiography while MRI remains the most sensitive modality for early disease detection.
